# Recommendations for healthy aging as documented by health professionals: a summative content analysis of health records

**DOI:** 10.1017/S1463423623000671

**Published:** 2024-01-09

**Authors:** Anna Nivestam, Maria Haak, Albert Westergren

**Affiliations:** Faculty of Health Sciences, Kristianstad University, Kristianstad, Sweden

**Keywords:** advice, health intervention, health promotion, older people, prevention

## Abstract

**Aim:**

To identify what type of recommendations were recorded in older adults’ health records by health professionals during preventive home visits.

**Background:**

To promote health and prevent ill health, health professionals can give support and recommendations to older adults. The preventive home visit for older adults is one example of an intervention where health professionals such as nurses, social workers, and assistant nurses can give recommendations. By exploring what recommendations are recorded and within what areas, we can also gain knowledge about areas where provision of recommendations seems lacking. This knowledge would provide health professionals with guidance in their counseling with the older adult.

**Methods:**

Records from preventive home visits (*n* = 596; mean age 78.71) were qualitatively and quantitatively analyzed.

**Findings:**

The most frequently recorded recommendations were related to *physical or mental illness*, *falls,* and then *nutrition*. The results showed that recommendations could be sorted into ten sub-categories related to *physical or mental illness*, *falls, nutrition, physical activity, preparation for the future, social participation, finances, getting help from others, municipal services*, and *security at home*. These ten sub-categories were classified into the International Classification of Functioning, Disability, and Health categories *body functions & structure* (including one sub-category), *activity* (including four sub-categories), *participation* (including three sub-categories), and *environmental factors* (including two sub-categories). From the results, we could conclude that the major focus was on risk prevention and less focus was on health promotion. Thus, the visitor’s recommendations most likely mirror the older adult’s explicit needs ‘here and now’ to a great extent. However, health visitors also need to focus on intrinsic capacities to promote health. Besides recommendations relating to the person’s intrinsic capacities, environmental aspects should be focused upon, to improve healthy aging.

## Introduction

To promote health and prevent ill health, health professionals can give support and recommendations to older adults. The preventive home visit for older adults is one example of an intervention where health professionals such as nurses, social workers, and assistant nurses can give recommendations (Tourigny *et al*., 2015).

In previous research, older adults described positive experiences, such as feeling recognized, feeling in control, and feeling prepared for the future, as consequences of the recommendations and support given during a preventive home visit (Nivestam *et al.,* 2020). However, knowing what recommendations health professionals record during a visit would be of interest. By exploring what recommendations are recorded and within what areas, we can also gain knowledge about areas where provision of recommendations seems lacking. This knowledge would provide health professionals with guidance in their counseling with the older adult.

Support and recommendations can be given to enable healthy aging. Theoretically, healthy aging has been defined by the World Health Organization (WHO) as ‘the process of developing and maintaining the functional ability that enables well-being in older age’ (WHO, 2015, p. 28). Functional ability is the essence of the definition, meaning that the person has the ability to do things they value, such as activities contributing to personal growth and maintenance of relationships. The person’s functional ability is determined by intrinsic capacities and environmental characteristics. Intrinsic capacities are the person’s mental and physical capacities, for example, the person’s ability to remember, move around, and talk. On the other hand, environmental aspects are, for example, the closest environment in the person’s home, outdoor spaces, buildings, and attitudes toward older adults. Therefore, to enable healthy aging, support and recommendation have to cover both intrinsic capacities and environmental aspects.

To support healthy aging and thereby people’s functional abilities, this study takes its point of departure in the International Classification of Functioning, Disability, and Health model (ICF model). The WHO describes the ICF model as a tool to assess, evaluate, and improve people’s functional ability (WHO, 2001). The ICF model has been used around the world both in research and practice for over 20 years (Cieza and Kostansjek, 2021). The model highlights different domains that interact with each other and affect a person’s functional ability. The domains are health conditions, body functioning & structures, activities, participation, environmental factors, and personal factors. Thus, both the ICF model (WHO, 2001) as well as the description of healthy aging (WHO, 2015) emphasize a complex interaction among different personal factors such as health conditions, how the body is functioning, what activities are performed and the environment around the person, all of which can improve or obstruct a person’s functional ability. It might be that an awareness of these domains could help health professionals in their decisions about what recommendations to give to older adults.

Electronic health records are usually used to document the health and care of patients in clinical settings and aggregated data from the records can also be used to identify areas that can support population health (Kruse *et al*., 2018). Reviewing the records can give a better understanding of what and how care is provided and thereby improve the care delivered (Birtwhistle and Williamson, 2015). Advantages have been seen with free text records in that much valuable information seems to be hidden in the texts, especially related to phenomena such as falls and malnutrition (Kharrazi *et al.*, 2018). Qualitative data seem to be important to get a deeper understanding of a complex phenomenon, such as health promotion and risk prevention. Therefore, it would be valuable to review the records made during a health intervention such as a preventive home visit to get new insights that could improve the recommendations given to older adults. The first aim of this study was to identify what type of recommendations were recorded in older adults’ health records by health professionals during preventive home visits. The second aim was to discover which recommendations were frequently recorded in relation to different characteristics, that is, men/women, having partner/being single, and subjective health.

## Methods

### Design

A qualitative and quantitative research design was used.

### Context

This study was conducted within the research and collaboration project Preventive home visits to seniors (Pre-H) (Nivestam, 2022). Within Pre-H, preventive home visits are offered with the purpose of promoting health and preventing ill health. Older adults (≥77 years) without home care are offered one visit by a health professional (nurse, district nurse, assistant nurse, or social worker). In total, nine visitors conducted preventive home visits in the seven municipalities included in the project. The visit lasted for approximately 1–2 h. During the visit, the visitor asked a set of comprehensive structured questions related to health and well-being. Examples of questions were about nutrition, falls, cognition, physical activity, anxiety, loneliness, activities in daily living, and the use of digital technology. Throughout the whole dialog, support and recommendations were given, with the purpose of motivating the older adult to act on the recommendations given. The visitor used a digital record system to record answers to the questions and recommendations given and were aware of that the information could be used in research. For more information about the project, see Nivestam (2022).

### Sample

Older adults’ records were drawn from the register used in Pre-H. From October 2018 to February 2020, all records where visitors had recorded some sort of recommendation were included, in total 596 people’s records. For an overview of characteristics from the older adults whose records were included, see Table 1.

**Table 1:** Sample characteristics

Characteristics	*n* = 596
Age, mean (SD)	78.71 (1.81)
Women *n* (%)	317 (53.4)
Men *n* (%)	277 (46.5)
Having a partner *n* (%)	387 (64.9)
Single *n* (%)	209 (35.1)
Subjective health *n* (%)	
Good	438 (73.6)
Bad	157 (26.4)

### Data collection

In the Pre-H register, the visitor can freely write, in their own words, any recommendation that they believe is appropriate for the older adult, and there is no limitation on the number of recommendations given. The recommendations are written in the digital support system at the end of the visit. In addition to the free text recorded recommendation, items described in Table 1 were extracted from the digital register. All sample characteristics described in Table 1 are rated by the older adult but recorded by the visitor. The item subjective health was dichotomized into good (excellent, very good, good) and bad (fair, bad) health.

### Analysis

Summative content analysis described by Hsieh and Shannon (2005) was used to analyze the records. To facilitate the sorting of the material, NVivo software was used. First, every recommendation registered in the records was given a code, and then, the codes were sorted into sub-categories with similar meanings. Thereafter, the frequencies of the codes in every sub-category were counted and summarized. In addition, the material was deductively sorted into categories inspired by the ICF model’s various domains, *body functions & structure*, *activity*, *participation,* and *environmental factors* (Socialstyrelsen, 2022). The ICF model also consists of the domains *health conditions* and *personal factors;* however, factors related to these domains were not classified in the ICF model. Therefore, these domains were not used for sorting the sub-categories. As a final step, in order to draw robust statistical conclusions, the most frequently recorded recommendations (>100 times) and given recommendations (coded as 0 = no recommendation given; and 1 = any recommendation(-s) given) were imported as items in SPSS (IBM SPSS statistics software version 27). Chi-square tests were used to make comparisons between men and women; partner and single; and bad and good health. *P*-value was set to <0.05.

### Ethical considerations

This study was approved by The Ethical Review Board, Lund, Sweden (reference number 2018/849 and 2020-02343). The visitors obtained informed consent from the participants before recording recommendations in the register. Data material transferred to the researcher was anonymous.

## Results

In the 596 health records, 873 specific recommendations were recorded. In addition, the visitors wrote ‘general recommendations’ 53 times, and 297 times they wrote ‘non-recommendation’ or ‘no’ (indicating that no specific recommendation was given). One to five recommendations were written in each person’s record. Out of the 1,223 notes written in relation to recommendations, 71 % comprised specific recommendations. The specific recommendations recorded formed ten sub-categories. One sub-category was sorted into the ICF category *body functions & structure*, four sub-categories into the ICF category *activity*, three sub-categories into the ICF category *participation,* and two sub-categories into the ICF category *environmental factors* (see Table 2). Recommendations belonging to the category *body functions & structure* were related to *physical or mental illness*, recommendations related to the category *activity* were about *falls, nutrition, physical activity, and preparation for the future*, recommendations related to the category *participation* were about *social participation, finances,* and *getting help from others*, and finally recommendations belonging to the category *environmental factors* were about *municipal services* and *security at home*. Out of the 873 specifically documented recommendations, the recommendations most frequently (>100 times) recorded were related to physical or mental illness (36%), followed by recommendations related to falls (26%), and nutrition (14%).

**Table 2.** The four sorting categories from the International Classification of Functioning, Disability, and Health model and their 10 sub-categories, number of times recommendations were recorded, and example of contents (*n* = 596)

Category	Sub-category *n* = 10	Recorded number of times *n* = 873^a^	Examples of contents
Body functions & structure	Recommendations related to physical or mental illness	310 times (36%)	The primary recommendation recorded was to go to the health care center. The health care center was most often recommended to check blood pressure, problems with incontinence, or hearing problems. Moreover, some people were recommended to visit the health care center due to problems with the skin, pain, cognition, dizziness, vision, tremors, anxiety, depression, impaired endurance, medication reconciliation, or blood sampling. Other recommendations related to the sub-category mental or physical illness were to visit the dentist or optician.
Activity	Recommendations related to falls	228 times (26%)	Use a non-slip mat, general falls preventive recommendations, do not climb on things, use stable shoes were most frequently recorded recommendations. Other recommendations were: use a stepladder, sit down when you get dressed, and remove carpets.
	Recommendations related to nutrition	124 times (14%)	Reduce time for night fasting, drink more, eat more often, eat more fruit and vegetables, and eat more protein were the most frequently reported recommendations. Other recommendations were eat in between meals and eat more regularly.
	Recommendations related to physical activity	29 times (3%)	Recommendations recorded about physical activities were related to taking walks, balance training, or going to the gym.
	Recommendations to prepare for the future	13 times (2%)	Housing queue (In Sweden, there is usually a housing queue system which one has to apply for in order to rent an apartment. The queuing system takes account of the time spent in the queue).
Participation	Recommendations related to social participation	19 times (2%)	Social recommendations recorded were about social activities arranged by the municipality, pension organizations, the church, or other organizations.
	Recommendations related to finances	21 times (2%)	Housing supplement (In Sweden, people ≥65 years who receive a full old-age pension can apply for a housing supplement from the Swedish Pensions Agency).
	Recommendations to get help from others	9 times (1%)	Cleaning help, help in the garden, help from relatives.
Environmental factors	Recommendations related to municipal services	72 times (8%)	Contact the municipality to get assistance aids such as a walker. Contact an occupational therapist, family caregiver adviser, a social worker, a physiotherapist, or a district nurse. Apply for transport service, senior housing, cleaning assistance, or housing adaptation.
	Recommendations related to security at home	48 times (6%)	The most common recommendations recorded were to have a telephone close to the bed at night, install a smoke alarm, invest in a fire blanket, and get a personal alarm.

a Excluded were notes recorded about ‘general recommendations’ written 53 times, and ‘non-recommendation’ or ‘no’ written 297 times.

The three sub-categories with the most frequently recorded recommendations (physical or mental illness, falls, and nutrition), and any recommendations given were compared between: men and women, having a partner and being single, and good and bad health (see Table 3). Older adults in bad health received a recommendation (86.0%) to a greater extent than those in good health (78.3%, *P*-value 0.038). Significantly more people in bad health received recommendations related to physical or mental illness (49.0%) than those in good health (33.6%, *P*-value <0.001). However, recommendations related to falls were significantly more often recorded among those in good health (33.8%) compared to those in bad health (22.3%, *P*-value 0.007). A non-significant difference was seen in the other items compared.

**Table 3.** The three recommendations that have been recorded >100 times, and any recommendation given, in relation to sex, partner/single, and subjective health (*n* = 596)

	Sex		*P*-value	Partner/single		*P*-value	Subjective health		*P*-value
	Women (*n* = 317)	Men (*n* = 277)		Having partner (*n* = 387)	Single (*n* = 209)		Good (*n* = 438)	Bad (*n* = 157)	
Recommendations related to physical or mental illness *n* (%)	123 (38.8)	101 (36.5)	0.557	140 (36.2)	85 (40.7)	0.178	147 (33.6)	77 (49.0)	**<0.001**
Recommendations related to falls *n* (%)	99 (31.2)	84 (30.3)	0.812	121 (31.3)	62 (29.7)	0.686	148 (33.8)	35 (22.3)	**0.007**
Recommendations related to nutritional aspects *n* (%)	51 (16.1)	46 (16.6)	0.865	59 (15.2)	38 (18.2)	0.354	67 (15.3)	30 (19.1)	0.267
Any recommendation given *n* (%)	252 (79.5)	225 (81.2)	0.596	313 (80.9)	166 (79.4)	0.670	343 (78.3)	135 (86.0)	**0.038**

A chi-square test was used to compare items, ***P*** **-value<0.05**

## Discussion

This study identified recommendations recorded by health professionals during preventive home visits in older adults’ health records and compared recommendations given to people with different characteristics. The results showed that recommendations could be sorted into ten sub-categories related to *physical or mental illness*, *falls, nutrition, physical activity, preparation for the future, social participation, finances, getting help from others, municipal services*, and *security at home*. These ten sub-categories were classified into the ICF categories *body functions & structure* (including one sub-category), *activity* (including four sub-categories), *participation* (including three sub-categories), and *environmental factors* (including two sub-categories). A significant difference was seen between older adults in bad health who were more often given recommendations related to physical or mental illness than those in good health. The opposite significant difference was shown related to recommendations about falls, which were more frequently given to people in good health compared to those with bad health.

Recommendations given to older adults living at home without home care seem to have a major focus on illness. A deficit-based approach seems to be dominant over the health-promotive perspective focusing on the person’s assets. Although the aim of preventive home visits in general is to prevent ill health and promote good health (Markle-Reid *et al.*, 2006; Tourigny *et al.*, 2015), the focus for most of the recommendations given seems to be on illness. The present results showed that 36% of the recommendations recorded were related to physical or mental illness. Recommendations related to physical or mental illness seem to target secondary prevention and mainly referral to health care centers to find a cure or treatment for an existing problem such as incontinence or cognitive decline. Traditionally in research, we would argue that a great focus has been on health deficits in older age (eg, García-Esquinas *et al*., 2019; Gordon *et al.*, 2019; Stolz *et al.*, 2021). However, a nuanced picture has to be added to the focus on health deficits in order to strengthen older adults’ assets (Hornby-Turner *et al*., 2017). For example, in addition to recommendations recorded, the visitor could have a dialog about the person’s assets as well as the deficits.

During preventive home visits, recommendations related to falls and nutrition are commonly recorded by health professionals. According to the results, 26% of the recommendations recorded were related to falls, and 14% were related to nutrition. Using a non-slip mat and reduced time for night fasting were most frequently recorded. It is not surprising that recommendations related to falls and nutrition are common. In research, falls (Peel *et al*., 2002) and malnutrition (Margetts *et al*., 2003; Martínez-Reig *et al*., 2014; Kupisz-Urbanska and Marcinowska-Suchowierska, 2022) are often mentioned as two major risk factors for ill health in older age. Moreover, much research can be found on how to prevent falls (Gillespie *et al.*, 2012; Sherrington *et al*., 2019) and malnutrition (Munk *et al*., 2016; Gusdal *et al*., 2021; Visser *et al*., 2022). However, just giving one recommendation, for example to use a non-slip mat, might not be enough. Fall prevention can involve multiple aspects (eg, home safety, physical activity, medication) (Gillespie *et al*., 2012), and therefore to really prevent the risk of falls, a care plan is recommended (WHO, 2017), or as we would rather call it, a prevention plan. The WHO (2017) highlights in their guidelines, *Integrated care for older people: Guidelines on community-level interventions to manage declines in intrinsic capacity,* appropriate approaches to assess, classify, and manage declines in physical and mental capacities. These guidelines can serve as an inspiration for preventive home visits. For example, the care plan that is recommended to manage a decline in intrinsic capacities (WHO, 2017) can be used during preventive home visits. During the preventive home visits, a comprehensive assessment is usually performed, and in addition to the assessment, it might be beneficial to create a more rigorous prevention plan in some cases where a decline in intrinsic capacities is found. In addition, the importance of maintaining or increasing one’s physical activity can be of importance to promote health.

To support healthy aging, the person’s intrinsic capacities are as important as environmental factors. Based on the present results, we could see that recommendations were focused on the person’s intrinsic capacities and their own behavior. By giving individual recommendations, there is a risk of overemphasizing the person’s own responsibility for behavioral changes and preventing poor health outcomes. To avoid placing the burden of achieving healthy aging solely on the individual, focus also must be on environmental factors. It is important not to ignore other determinants of health, such as finances and family support, that may reduce or support the capacity of the older adult to act on the recommendations.

To systematize, facilitate, and improve the recording of recommendations during the preventive home visit, inspiration can be taken from the ICF classification system. The present results showed that the recommendations recorded cover the domains in the ICF model: *body functions & structure, activity, participation,* and *environmental factors*. Using the ICF model (WHO, 2001) as a guide for recording and counseling might ensure that the visitor also reflects upon factors that can improve or maintain a person’s functional ability and does not focus only on recommendations related to *body function & structure*. However, to optimize healthy aging more focus has to be on environmental factors outside one’s home. Environmental factors are emphasized both in the ICF model (WHO, 2001) and WHO’s theoretical model for healthy aging (WHO, 2015). A great focus during the preventive home visits seems to be on intrinsic capacities since 36% of the recommendations given were related to *body functions & structure*. The *environmental factors* (14%) addressed in the recorded recommendations seem to focus on municipal services provided at home and security in one’s own home. Therefore, to promote healthy aging during the preventive home visits it would be beneficial to also discuss environmental aspects outside one’s home. For example, the visitor can give recommendations about how to overcome obstacles experienced in the immediate environment. In addition, the public environment has to be adapted to older adults’ needs. One way of doing this could be to use the records from the preventive home visits as a basis for taking societal decisions and thereby improving the environment (Nivestam *et al.,* 2021). Thus, systematizing the recorded recommendations could give an indication of older adults’ needs and thereby guide policymakers in societal decisions regarding the public environment.

In addition to the public environment, it could be beneficial for policymakers to understand older adults’ needs in their home environment. For example, data on recommendations could be used to support a claim for additional in-home support to enable older adults to act on the recommendations they have been provided with from the home visitor. A dialog between policymakers and home visitors seems to be essential to promote health aging, to be able to take actions both on an individual and a societal level.

Another domain described in the ICF model, which affects a person’s functional ability, is participation. In the present study, only 5% of the recommendations were categorized as *participation* and 2% were related to *social participation*. This could be seen as a strength in that older adults may not need such recommendations. Or it could be seen as a weakness in that the visitors did not highlight the importance of social participation. In order to give more comprehensive recommendations that supports older adults’ functional ability and thereby health, recommendations about social participation should be reflected upon. These results also give incentive for using the ICF model as a guide when giving recommendations to support functional ability.

We now move on to methodological considerations worth discussing. First, this is a study based on records, and we do not know anything about other recommendations given during the dialog, which might not be recorded. In addition, what is recorded might mirror not only the older adult’s actual needs but also the home visitor’s actual focus. The visitor’s profession or specific education could have an impact on what is recorded; this would be interesting to investigate in future research. The recommendations are recorded at the end of the dialog, which might have an impact on the number of recommendations recorded as well as the focus of the recommendations. Furthermore, the fact that the visitors know that information recorded in the digital record system could be used in research could potentially affect what they record. This could lead to the visitor omitting some information; however, it could also result in more accurate documentation, due to that they know someone else will investigate what is written. There can also be a tiring effect at the end of the visit, which may lead the visitor to record less recommendations than given. Moreover, some records just contained a note about ‘general recommendations’ or ‘non-recommendation’ given. These records were difficult to interpret, and one can question the purpose or usefulness of recording this type of ‘general recommendations’. The visitors recorded ‘non-recommendation’ or ‘no’ 297 times, and we could see in our results that people in bad health were given recommendations to a larger extent than those in good health. In addition, some recommendations might fit multiple sub-categories, for example, to engage in balance training was sorted into the sub-category ‘recommendations related to physical activity’; however, recommendation about engaging in balance training could also be related to falls and thereby sorted into that sub-category.

## Conclusion

Recommendations recorded in older adults’ health records during preventive home visits focus on physical or mental illness, falls, and nutritional aspects. Thus, the major focus was on risk prevention and less focus was on health promotion. To increase the health-promotive focus during preventive home visits, the records could be guided by the ICF model. The ICF model could guide the visitor in giving more comprehensive recommendations and can help ensure that important aspects are not missed. It would therefore be favorable to include the ICF model when educating home visitors. Moreover, in some cases where risks are detected during the comprehensive assessment made during the visits, a special care plan, or rather a prevention plan, might be needed. With a prevention plan, it can be possible to monitor and evaluate the development of a person’s intrinsic capacities and thereby enable healthy aging. In addition, to enable healthy aging, the focus has to be on both intrinsic capacities and environmental aspects. Recommendations related specifically to environmental aspects might be needed. This could facilitate older adults’ participation in society and thereby allow them to improve environmental aspects that would contribute to healthy aging.
